# The genetic architecture of fornix white matter microstructure and their involvement in neuropsychiatric disorders

**DOI:** 10.1038/s41398-023-02475-6

**Published:** 2023-05-26

**Authors:** Ya-Nan Ou, Yi-Jun Ge, Bang-Sheng Wu, Yi Zhang, Yu-Chao Jiang, Kevin Kuo, Liu Yang, Lan Tan, Jian-Feng Feng, Wei Cheng, Jin-Tai Yu

**Affiliations:** 1 Department of Neurology, Qingdao Municipal Hospital, Qingdao University, Qingdao, China; 2 Department of Neurology and Institute of Neurology, Huashan Hospital, State Key Laboratory of Medical Neurobiology and MOE Frontiers Center for Brain Science, Shanghai Medical College, Fudan University, National Center for Neurological Disorders, Shanghai, China; 3 Institute of Science and Technology for Brain-Inspired Intelligence, Fudan University, Shanghai, China; 4 Key Laboratory of Computational Neuroscience and Brain-Inspired Intelligence (Fudan University), Ministry of Education, Shanghai, China; 5 Fudan ISTBI—ZJNU Algorithm Centre for Brain-Inspired Intelligence, Zhejiang Normal University, Jinhua, China; 6 MOE Frontiers Center for Brain Science, Fudan University, Shanghai, China; 7 Zhangjiang Fudan International Innovation Center, Shanghai, China

**Keywords:** Neuroscience, Diseases

## Abstract

The fornix is a white matter bundle located in the center of the hippocampaldiencephalic limbic circuit that controls memory and executive functions, yet its genetic architectures and involvement in brain disorders remain largely unknown. We carried out a genome-wide association analysis of 30,832 UK Biobank individuals of the six fornix diffusion magnetic resonance imaging (dMRI) traits. The post-GWAS analysis allowed us to identify causal genetic variants in phenotypes at the single nucleotide polymorphisms (SNP), locus, and gene levels, as well as genetic overlap with brain health-related traits. We further generalized our GWAS in adolescent brain cognitive development (ABCD) cohort. The GWAS identified 63 independent significant variants within 20 genomic loci associated (*P* < 8.33 × 10^−9^) with the six fornix dMRI traits. *Geminin coiled-coil domain containing* (*GMNC*) and *NUAK family SNF1-like kinase 1* (*NUAK1*) gene were highlighted, which were found in UKB and replicated in ABCD. The heritability of the six traits ranged from 10% to 27%. Gene mapping strategies identified 213 genes, where 11 were supported by all of four methods. Gene-based analyses revealed pathways relating to cell development and differentiation, with astrocytes found to be significantly enriched. Pleiotropy analyses with eight neurological and psychiatric disorders revealed shared variants, especially with schizophrenia under the conjFDR threshold of 0.05. These findings advance our understanding of the complex genetic architectures of fornix and their relevance in neurological and psychiatric disorders.

## Introduction

The fornix is a C-shaped white matter bundle located in the center of the hippocampaldiencephalic limbic circuit [1]. It is the predominant efferent tract connecting the hippocampus to the mammillary bodies, thalamic nuclei, and prefrontal cortex [2], and mainly controls the formation of spatial memory, episodic memory, and executive functions [3]. Memory impairments were significantly related to fornix diffusion tensor imaging (DTI) traits in previous studies [4]. Using in vivo and in vitro magnetic resonance imaging (MRI) data, and mice models, researchers have strengthened the hypothesis that the fornix plays a role in Alzheimer’s disease (AD), and can be used as a disease biomarker and a therapeutic target [5]. In addition, one DTI study further demonstrated a disruption in fornix integrity in male patients with schizophrenia (SCZ) [6]. This suggests that the fornix is not only involved in memory-related brain disorders but may also be involved in the development of psychiatric disorders. Nonetheless, as most studies have imaged or stimulated large areas of the fornix, it is currently unclear the genetic architecture of the fornix and its involvement in these brain disorders.

Parameters from diffusion MRI (dMRI) techniques, such as DTI and neurite orientation dispersion and density imaging (NODDI) can lead to a better understanding of the fornix microstructure. DTI permits in vivo assessment of neural microstructure by utilizing the diffusion properties of water in constrained compartments and can provide a measure of the coherence of neuronal fibers [7]. The two most commonly studied metrics are fractional anisotropy (FA) and mean diffusivity (MD). FA describes the directional diffusivity of water along the fiber bundle, which reflects the fiber integrity [8]. Changes in FA closely reflect altered myelination or demyelination [9]. MD represents the overall diffusivity in a given voxel, with lower values indicating less diffusivity [8]. Several studies of diffusivity within the human fornix have identified changes that occur with normal aging [10] and in neurodegenerative diseases [11]. FA decreases with aging while MD increases, possibly reflecting degradation, breakdown, or deterioration in fiber integrity [12]. Moreover, the more complex models like NODDI are contributing additional information on different diseases at the clinical level. NODDI provides orientation dispersion index (OD), intra-cellular volume fraction (ICVF), and isotropic or free water volume fraction (ISOVF) maps that reflect the morphology of axons and dendrites and their branching complexity [13]. OD could detect water diffusivity. The higher ISOVF indicated increased extracellular water volume, expected in neuroinflammatory states [14].

**Fig. 1:**
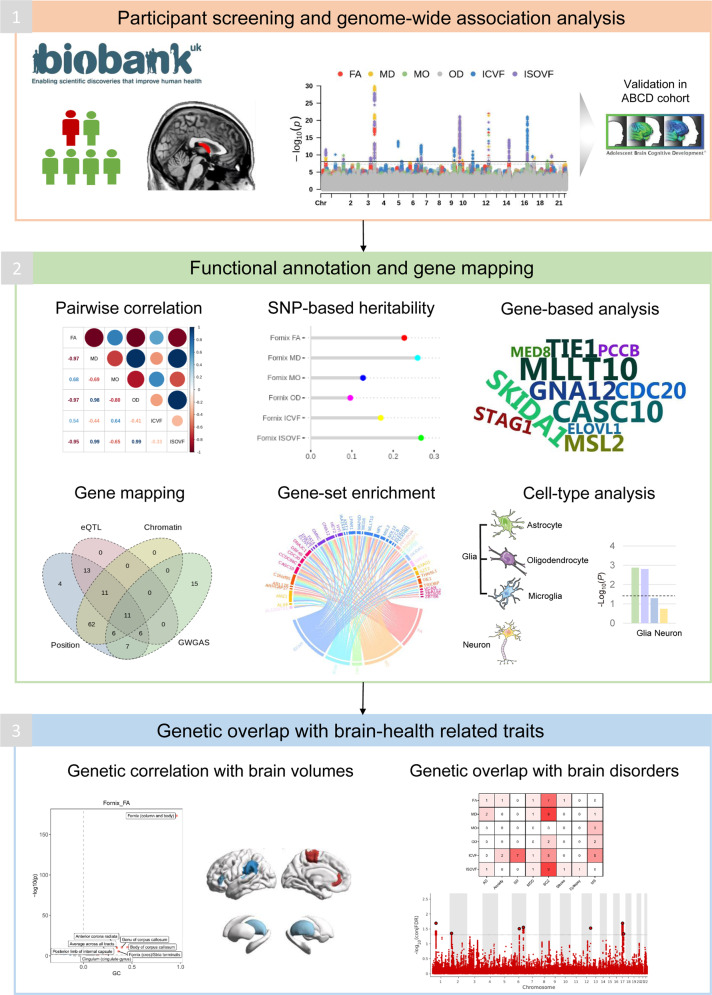
The analytical workflow of the study. GWAS genome-wide association study, ABCD adolescent brain cognitive development; PC principal component, SNP single nucleotide polymorphism, FA fractional anisotropy, MD mean diffusivity, MO diffusion tensor mode, OD orientation dispersion index, ICVF intra-cellular volume fraction, ISOVF isotropic or free water volume fraction.

A comprehensive knowledge of the forniceal white matter microstructure could serve to inform clinicians of cognitive subpathways and corresponding memory deficits that arise from neurodevelopmental and neurodegenerative diseases. However, the genetic underpinnings of the fornix region remain underexplored. Here, we aimed to: (1) illuminate the genetic architecture of the fornix white matter microstructure using three DTI models (FA, MD, and MO) and three NODDI models (OD, ICVF, and ISOVF) by performing the first and largest genome-wide association study (GWAS) to date; (2) and investigate its involvement in common brain disorders, especially those reported in previous observational studies. The analytical workflow of this study is shown in Fig. 1.

## Materials and methods

### Participants description and quality control procedures

We used the brain dMRI data from 41,984 genotyped individuals from the UK Biobank (UKB) (http://www.ukbiobank.ac.uk/) [15] under accession number 19542. UKB has received ethical approval from the National Health Service National Research Ethics (ref: 11/NW/0382) and obtained informed consent from its participants. The generalization sample comprised dMRI and single nucleotide polymorphisms (SNP) data from the Adolescent Brain Cognitive Development (ABCD) cohort collected from 21 acquisition sites [16]. The ABCD study was approved by the Institutional Review Board (IRB) of the University of California San Diego (IRB# 160091) and all of the study sites obtained approval from their local IRBs. Parents or guardians provided written informed consent, and children assented before participation. The present study was conducted in accordance with the Declaration of Helsinki.

Our analytic sample was restricted to white British individuals whose data were used in calculating principal components (PCs). We applied standard quality control (QC) procedures to the UKB v3 imputed genetic data by removing SNPs with call rate < 0.95, imputation quality score < 0.5, a minor allele frequency (MAF) < 0.01, failing the Hardy–Weinberg equilibrium tests at *P* < 1 × 10^–06^, or duplicated, and further filtered out individuals with abnormal sex chromosome, putative sex chromosome aneuploidies, heterozygosity rate outliers, having more than 10 putative third-degree relatives, or missing genotype rate ≥ 5% using PLINK [17]. For ABCD, we downloaded the genetic data from the third release and subsequently applied similar post-imputation QC procedures. After QC, the final analytic sample size in UKB and ABCD were 30,832 and 3613, respectively. The mean intraclass correlation coefficients (ICCs) were excellent for all of the six phenotypes (ICC = 0.813–0.972) in UKB (Table S1). Comparatively speaking, the ICCs were poor but relatively acceptable (ICC = 0.303–0.490) in ABCD.

### Genetic association analyses and identification of genomic loci

GWAS was run via PLINK 1.9 [17] adjusting for age, age^2^, sex, scanning site, intracranial volume (ICV), and the first 10 genetic PCs. We first performed univariate GWAS of the individual traits, then the resulting residuals for the 6 traits were jointly fed into the multivariate omnibus statistical test (MOSTest) analysis [18]. MOSTest implements permutation testing to identify genetic effects across multiple phenotypes [19], yielding a multivariate GWAS summary statistic across all six features.

Genomic risk loci were identified using the Functional Mapping and Annotation (FUMA) of the GWAS SNP2GENE online platform [20] (version 1.3.7, http://fuma.ctglab.nl/). Allele linkage disequilibrium (LD) correlations were computed from the European panel of the 1000 Genomes phase 3 data. Independent significant SNPs were identified by the statistical threshold (5 × 10^–08^/6 = 8.33 × 10^–09^) and independency (*r*^2^ ≤ 0.6) [21]. Lead variants were defined as those significant variants that are independent of each other at *r*^2^ < 0.1 [21]. Candidate SNPs were defined as all SNPs in LD (*r*^2^ ≥ 0.6) with one of the independent significant SNPs in the genetic loci [21]. Genomic risk loci were characterized by merging LD blocks that are located close to each other (<250 kb apart) [21].

The NHGRI-EBI GWAS catalog [22] was subsequently searched for independent significant SNPs and relevant SNPs (SNPs in LD with them) to look for reported associations with any other traits. We mainly focused on traits related to brain imaging, cognitive functions (e.g., general cognitive ability), neurodegenerative diseases (e.g., AD), and neuropsychiatric disorders (e.g., depression, SCZ, and bipolar disoder [BD]).

### SNP-based heritability

SNP-based heritability analyses were conducted using linkage disequilibrium score (LDSC) regression [23]. Heritability describes the proportion of phenotypic variance explained by genetic variance, in which genomic inflation factors (λ_GC_), LDSC intercepts, and LDSC ratios for each GWAS were calculated. We used precomputed LD scores calculated by 1000 Genomes European data. In addition, we calculated the pairwise genetic correlation estimates between the six fornix phenotypes using LDSC v1.0.1.

### Gene mapping, gene-based association, and gene-set analysis

FUMA [20] annotates significantly fornix-linked SNPs with functional categories, including Combined Annotation-Dependent Depletion (CADD) scores [24], RegulomeDB scores [25], and 15-core chromatin states [20], using a hypergeometric test. A CADD score above 12.37 is suggestive of a deleterious protein effect [24], whereas a lower RegulomeDB score indicates a higher probability of regulatory function. Categories 1–7 of chromatin states are considered open chromatin states [26]. Positional, expression Quantitative Trait Loci (eQTL), and 3D chromatin interaction mappings [20] were used to map all of the independent significant variants to genes. Positional mapping was used to map SNPs to protein-coding genes based on the physical distance within 10 kb in the human reference assembly (GRCh37/hg19). eQTL was used to map SNPs to genes when they are associated with variation in gene mRNA expression levels. Chromatin interaction mapping was performed to map SNPs to genes based on brain-associated Hi-C chromatin conformation capture datasets. We used default values for all of the parameters and applied an false discovery rate (FDR) of 0.05 to define significant associations.

Genome-wide gene-based association analysis (GWGAS) was performed using GWAS summary statistics as input into multimarker analysis of genomic annotation (MAGMA) (v1.08) [27] with default settings, which made use of the European panel of the 1000 Genomes phase 3 data as the reference. The major histocompatibility complex (MHC) region was excluded before the analysis. The Bonferroni-corrected significant threshold was *P* = 0.05/18,879 genes = 2.65 × 10^–06^. In addition, we performed a gene-set analysis using the g:Cocoa (compact comparison of annotations) function in g:Profiler web server for curated gene sets and Gene Ontology (GO) terms.

### Cell specificity analysis

To assess whether genes are disproportionately expressed in certain cell types, we investigated associations with several gene expression profiles using MAGMA’s gene expression analysis. We used the CELL TYPE function in FUMA to test whether fornix-linked genes were associated with differential expression levels across different cell types. FDR-corrected *P* values < 0.05 were considered significant.

### Genetic correlations with other DTI and brain volumetric phenotypes

We used LDSC [23] to estimate the pairwise genetic correlations (*rg*) between fornix traits and 22 DTI phenotypes reported by Zhao et al.’s GWAS [28]. For the brain cortical phenotypes, we chose Grasby et al.’s GWAS [29], who reported the cortical area and thickness using the ENIGMA database. For the subcortical phenotypes, we utilized Hibar et al.’s GWAS [30]. Genetic correlations for which the *P* values survived the FDR correction (*P* < 0.05) were considered significant.

### Genetic overlap between fornix white matter and brain health-related traits

To further examine the genetic overlap between fornix phenotypes and 10 brain health-related traits, GWAS summary statistics for cognitive traits (AD [31] and reaction time [32]), psychiatric traits (anxiety disorders [33], BD [34], depression [35], and SCZ [36]), vascular traits (stroke [37] and white matter hyperintensities [WMH] [38]), and others (epilepsy [39] and multiple sclerosis (MS) [40]) were obtained. LDSC regression analyses were applied to detect the genetic correlations.

Further cFDR (condFDR and conjFDR) methods using MATLAB R2018b and Python 3.7.7 were employed to investigate the shared loci between fornix phenotypes and the eight brain disorders. Using the associations between genetic variants and the secondary phenotype, the condFDR analysis re-ranked test statistics and recalculated the associations between these variants and the primary phenotype, thus prioritizing variants for follow-up analyses [41]. We plotted the empirical cumulative distribution of nominal *P* values for all SNPs in one phenotype (e.g., fornix FA) and for subsets of SNPs with significance levels in another phenotype (e.g., SCZ) below the indicated cutoffs (*P* ≤ 1, *P* ≤ 0.1, *P* ≤ 0.01, and *P* ≤ 0.001). The enrichment is visualized as successive leftward deflections from the null distribution in conditional Quantile–Quantile (Q–Q) plots. All *P* values were corrected for inflation using a genomic inflation control procedure, considering that the empirical null distribution in GWASs is affected by global variance inflation [41]. We next made use of the conjFDR [42] method, which is an extension of condFDR and defined by the maximum of the two condFDR values for a specific SNP, to detect the genetic loci shared between traits. This method estimates a posterior probability that an SNP is null for either or both traits at the same time, given that the *P* values for that SNP are lower than the observed *P* values in both the primary and secondary phenotypes. Regions of complex LD patterns, such as MHC (chr 6: 25119106–33854733) and 8p23.1 (chr 8: 7242715–12483982) regions, apolipoprotein E (APOE) for AD, and microtubule-associated protein tau (MAPT) for Parkinson’s disease (PD) were excluded before performing the analysis. The FDR significance cutoffs were 0.01 for condFDR and 0.05 for conjFDR, in line with prior studies [43].

## Results

### GWAS results of fornix white matter microstructure

**Fig. 2:**
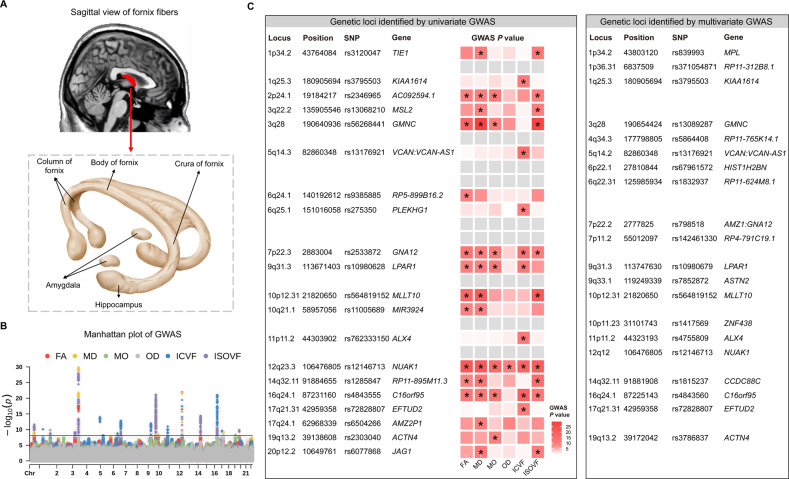
The comparison of genetic loci between the univariate and multivariate GWAS. **A** The location of the fornix in the human brain (colored in red) and the subdivisions from the sagittal view. **B** The Manhattan plot of genetic variants underlying univariate GWAS of the fornix. Different colors represent different phenotypes, with the horizontal red line denoting GWA significance (*P* < 8.33 × 10^–09^). **C** The comparison of genetic loci between the univariate and multivariate GWAS. The left column indicates the 20 genetic loci identified by the univariate GWAS, whereas the right column indicates the 20 loci identified by the multivariate GWAS. The heatmap shows the significant genetic loci for, from left to right, FA, MD, MO, OD, ICVF, and ISOVF. Significant loci in the univariate GWAS (*P* < 8.33 × 10^–09^) are marked with an asterisk. 11 of the 20 loci identified in the univariate GWAS were replicated in the multivariate GWAS. GWAS genome-wide association study, FA fractional anisotropy, MD mean diffusivity, MO diffusion tensor mode, OD orientation dispersion index, ICVF intra-cellular volume fraction, ISOVF isotropic or free water volume fraction, SNP single nucleotide polymorphism.

The GWAS made use of data from 30,832 UKB brain imaged samples (47.1% females; age range: 40–70 years; Table S2), with six fornix phenotypes (FA, MD, MO, OD, ICVF, and ISOVF), accounting for age, age^2^, sex, imaging site, ICV, and the first 10 genetic PCs. The location of the fornix region in the human brain and the fiber bundles and their partitions were depicted in Fig. 2A.

The univariate GWAS identified significant hits for all of the six phenotypes after correcting for the number of traits analyzed (Bonferroni-corrected *P* < 5 × 10^–08^/6 = 8.33 × 10^–09^) and a total of 63 independent significant SNPs, tagging 20 independent genomic loci (Fig. 2B, Tables S3, S4). The Q–Q plots depicted that potential population stratification and/or cryptic relatedness are well controlled after genomic correction (Fig. S1). Specifically, 10 of the 20 genetic loci were associated with fornix FA; 13, 7, 2, 10, and 10 loci were associated with MD, MO, OD, ICVF, and ISOVF of the fornix, respectively. Five of the 20 genetic risk loci were associated with only one trait; within 4 of them were associated with ICVF and one with FA. The subsequent multivariate GWAS identified a total of 20 genetic loci and a total of 44 independent significant SNPs under the *P* < 5 × 10^–08^ (Tables S4, S5). Overall, 11 of the 20 loci identified in the multivariate GWAS were replicated in the univariate GWAS, leading to a total of 29 unique genomic loci associated with fornix phenotypes (Fig. 2C).

### Generalization in ABCD cohort

The fornix-associated significant SNPs of the UKB sample were further evaluated in the generalization GWAS of the ABCD cohort (*N* = 3,613; 47.0% females; age range: 9–11 years), in which only the data on FA and MD were available. We found that 7 out of the 20 independent significant SNPs for FA discovery GWAS had the same effect direction in the generalization, and 14 of 25 for MD GWAS (Table S3). Moreover, 4 of the discovery lead SNPs had uncorrected *P* < 0.05, whereas 21 had uncorrected *P* > 0.05 in the generalization cohort for MD. The *Geminin coiled-coil domain containing* (*GMNC*) and *NUAK family SNF1-like kinase 1* (*NUAK1*) gene were found in UKB (*P* = 1.30 × 10^–30^ for *GMNC; P* = 3.98 × 10^–22^ for *NUAK1*) and replicated in ABCD (*P* = 1.15 × 10^–04^ for *GMNC*; *P* = 5.26 × 10^–03^ for *NUAK1*).

### Functional annotation and gene-based association

**Fig. 3:**
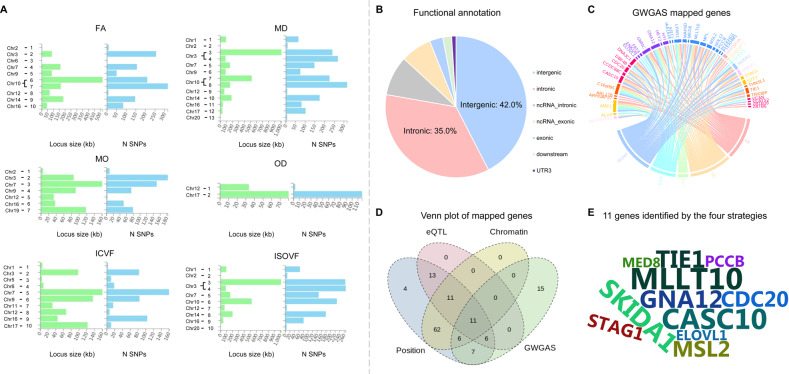
Functional annotation and gene mapping. **A** Overview of the genomic loci sizes and a number of variants. **B** Distribution of functional consequences of SNPs in significant genomic loci. **C** The genes identified by GWGAS for the five fornix phenotypes (FA: pink, MD: orange, MO: green, ICVF: blue, ISOVF: cyan). **D** Venn diagram of the number of genes mapped by the four different strategies, i.e., positional (green), eQTL (blue), chromatin interaction mapping (yellow), and identification by the GWGAS (red). A total of 213 genes were identified by all four approaches. **E** Seven genes were identified by all of the four mapping strategies, showed by the wordcloud plot. SNP single nucleotide polymorphism, FA fractional anisotropy, MD mean diffusivity, MO diffusion tensor mode, OD orientation dispersion index, ICVF intra-cellular volume fraction, ISOVF isotropic or free water volume fraction, eQTL expression quantitative trait loci, GWGAS genome-wide gene-based association analyses.

We mapped SNPs to genes via positional, eQTL, and chromatin interaction strategies in FUMA [20] (Tables S6–S8). The size of each of the locus and the number of mapped genes are shown in the Fig. 3A. Positional mapping showed that a majority of these independent significant SNPs were significantly enriched for noncoding regions, i.e., 42.0% for intergenic, and 35.0% for intronic (Fig. 3B, Table S3). About 87.0% of the SNPs had a minimum chromatin state of 1–7, indicating a location within regulatory regions. CADD scores indicated 3 SNPs (rs12146713, rs140589730, and rs62056161) as pathogenic with scores > 12.37.

GWGAS was performed by MAGMA [27], and 45 unique genes (*P* < 2.65 × 10^–06^) were detected (Table S9). No genes were identified for OD; the mapped genes of the rest five white matter traits are shown in Fig. 3C. In summary, these four strategies identified a total of 135 unique genes, where 19 were implicated by one mapping strategy, 75 genes by two strategies, 30 by three strategies, and 11 by all of the four types of gene mapping (Fig. 3D). The list of the 11 genes was depicted in the wordcloud plot (Fig. 3E). For example, the indicated *GNA12*, falling into the Gprotein subfamilies of G12 and Gq, has been indicated to negatively regulate cell adhesion [44]. It has been reported to play a key role in the genetic architecture underlying normal gray matter density variation in frontal and parietal regions [45] and was also a risk gene for SCZ [46].

Functional enrichment analysis identified 9 GO sets significantly associated with fornix traits (*P* < 0.05) using g:Profiler web server (Table S10). Overall, we found three associations with “cell development and differentiation”-related sets, including “cell development (GO:0048468), *P* = 7.77 × 10^–03^”, “cell differentiation (GO:0030154), *P* = 0.012”, and “cellular developmental process (GO:0048869), *P* = 0.014” pathways.

### SNP-based heritability and pairwise correlation

**Fig. 4:**
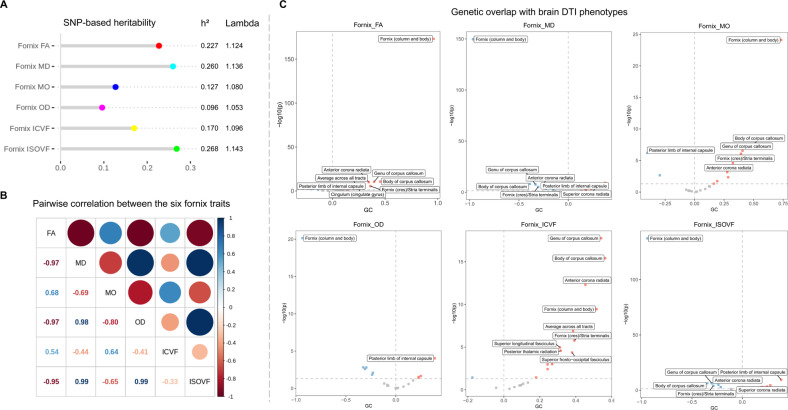
Heritability estimates and genetic overlap with other DTI traits. **A** LDSC-based heritability estimate for the six fornix phenotypes. All traits were significantly heritable, with heritability estimates ranging from 10% for OD to 27% for ISOVF. **B** Pairwise correlation matrix between the six fornix traits. These six traits were moderately to highly correlated, ranging from −0.33 to 0.99. **C** Volcano plots visualize the genetic correlation estimates between the six fornix traits and other DTI traits. Red, blue, and grey dots indicate positive, negative, and non-significant genetic associations, respectively. Correlation estimates that survived FDR adjustment (*P*_FDR_ < 0.001) are annotated with brain region names. SNP single nucleotide polymorphism, LDSC linkage disequilibrium score, DTI diffusion tensor imaging, FA fractional anisotropy, MD mean diffusivity, MO diffusion tensor mode, OD orientation dispersion index, ICVF intra-cellular volume fraction, ISOVF isotropic or free water volume fraction, FDR false discovery rate.

SNP-based heritability (*h*^2^) was 23% for FA, 26% for MD, 13% for MO, 10% for OD, 17% for ICVF, and 27% for ISOVF, illustrating themselves as genetically determined traits (Fig. 4A, Table S11). The pairwise correlation results indicated that these six indicators were moderate to highly correlated, ranging from −0.33 for ICVF and ISOVF to 0.99 for MD/OD and ISOVF (Fig. 4B; Table S12).

### Concordance with previous studies

Association lookups for the independent significant variants in the NHGRI-EBI GWAS catalog [22] were performed (Table S13). 15 of these 20 loci were previously reported to be related to brain volumes and white matter microstructure traits. We also noted that 10p12.31 was related to education attainment and 3q22.2 with cognitive performances. However, 4 of these 20 loci have not been reported to be related to imaging or cognitive/psychiatric traits before, namely the loci of 6q24.1, 10q21.1, 11p11.2, and 17q21.31.

### Genetic overlap with brain DTI and volumetric traits

We used the GWAS results to estimate the genetic overlap with brain DTI and volumetric traits via LDSC [23]. A total of 10, 12, 10, 8, 12, and 11 brain DTI traits were associated with FA, MD, MO, OD, ICVF, and ISOVF, respectively (*P*_FDR_ < 0.05, Table S14; associations with *P*_FDR_ < 0.001 were shown in Fig. 4C). For example, positive significant genetic correlations with the column and body of fornix (*rg* = 0.96, *P*_FDR_ = 9.94 × 10^–172^), the body of corpus callosum (*rg* = 0.45, *P*_FDR_ = 3.26 × 10^–10^), genu of corpus callosum (*rg* = 0.40, *P*_FDR_ = 4.84 × 10^–10^), anterior corona radiata (*rg* = 0.34, *P*_FDR_ = 1.07 × 10^–09^), fornix (cres)/stria terminalis (*rg* = 0.36, *P*_FDR_ = 1.37 × 10^–05^) and cingulum (*rg* = 0.28, *P*_FDR_ = 3.38 × 10^–04^), and reverse correlation with the posterior limb of the internal capsule (*rg* = −0.28, *P*_FDR_ = 1.05 × 10^–04^) for fornix FA were identified.

**Fig. 5:**
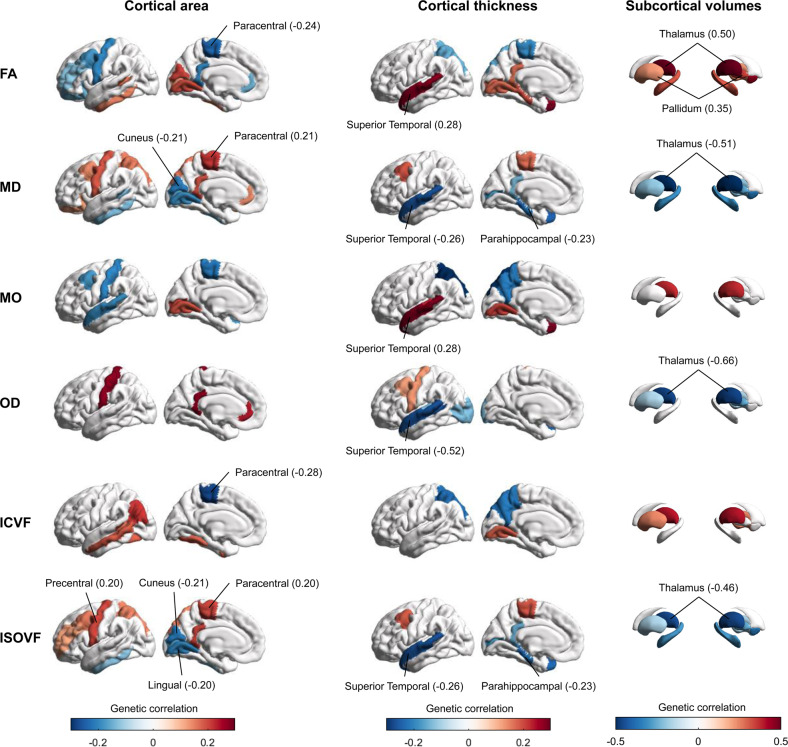
Genetic relationships with brain volumetric phenotypes. Different MRI brain volumetric structures were shown here, with cortical area (left panel), cortical thickness (middle panel), and subcortical volumes (right panel). Warm and cool colors indicate positive and negative associations, respectively. Significant correlations were annotated with brain region names (passed the two-sided *P*_FDR_ < 0.05). FA fractional anisotropy, MD mean diffusivity, MO diffusion tensor mode, OD orientation dispersion index, ICVF intra-cellular volume fraction, ISOVF isotropic or free water volume fraction, FDR false discovery rate.

Most of the six fornix traits were reported to be related with the area of paracentral (*rg* = −0.24, *P*_FDR_ = 0.025 for FA; *rg* = 0.21, *P*_FDR_ = 0.029 for MD; *rg* = −0.28, *P*_FDR_ = 0.023 for ICVF; *rg* = 0.20, *P*_FDR_ = 0.042 for ISOVF) and cuneus (*rg* = −0.21, *P*_FDR_ = 0.029 for MD; *rg* = −0.21, *P*_FDR_ = 0.023 for ISOVF), the thickness of superior temporal (*rg* = 0.28, *P*_FDR_ = 2.56 × 10^–03^ for FA; *rg* = −0.26, *P*_FDR_ = 2.56 × 10^–03^ for MD; *rg* = 0.28, *P*_FDR_ = 0.029 for MO; *rg* = −0.52, *P*_FDR_ = 3.49 × 10^–04^ for OD; *rg* = −0.26, *P*_FDR_ = 2.56 × 10^–03^ for ISOVF), and parahippocampal (*rg* = −0.23, *P*_FDR_ = 8.40 × 10^–03^ for MD; *rg* = −0.23, *P*_FDR_ = 5.13 × 10^–03^ for ISOVF), and thalamus (*rg* = 0.50, *P*_FDR_ = 4.22 × 10^–03^ for FA; *rg* = −0.51, *P*_FDR_ = 2.56 × 10^–03^ for MD; *rg* = −0.65, *P*_FDR_ = 8.40 × 10^–03^ for OD; *rg* = −0.46, *P*_FDR_ = 5.13 × 10^–03^ for ISOVF; Table S15, Fig. 5).

### Cell-type analysis

**Fig. 6:**
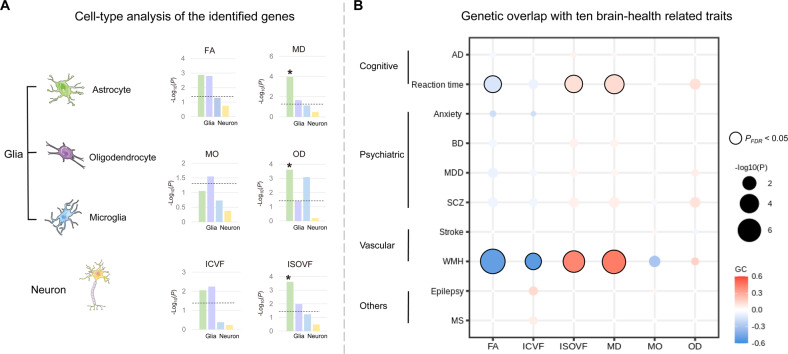
Cell-type analysis of the genes and their overlap with common brain-health-related traits. **A** Cell-type analysis indicated that most of the genes were significantly enriched in astrocytes. The *x*-axis of the histogram represents the cell types (green: astrocyte, purple: oligodendrocyte, blue: microglia, orange: neuron) and the *y*-axis represents the −log_10_(*P*), with the horizontal dotted line denoting the FDR significance (*P*_FDR_ < 0.05). Significant cell-type enrichments are marked with an asterisk. **B** Genetic correlations between fornix and ten brain-health-related traits were assessed using LDSC regression. Warm and cool colors indicate positive and negative associations, respectively. Significant positive correlations with reaction time and WMH were revealed, as indicated by a black frame (passed the two-sided *P*_FDR_ < 0.05). FA fractional anisotropy, MD mean diffusivity, MO diffusion tensor mode, OD orientation dispersion index, ICVF intra-cellular volume fraction, ISOVF isotropic or free water volume fraction, AD Alzheimer’s disease, BD bipolar disorders, MDD major depression disorders, SCZ schizophrenia, WMH white matter hyperintensities, MS multiple sclerosis, FDR false discovery rate.

We determined the neuronal cell types to which gene signals of fornix white matter were significantly enriched via LDSC [23] (Table S16). We found significant evidence of association for astrocytes (PsychENCODE_Adult database, MD: *P*_FDR_ = 0.022; OD: *P*_FDR_ = 0.036; ISOVF: *P*_FDR_ = 0.036; Fig. 6A). Astrocytes are the most abundant cell type in the brain, playing vital roles in governing key steps in synapse formation and plasticity [47].

### Genetic overlap with brain-health-related disorders

LDSC regression [23] was used to estimate the overlap between the genetic architectures of fornix and ten brain-health-related traits (Fig. 6B; Table S17). Overall, three traits showed significant consistent genetic correlations across the genome with reaction time (FA: *rg* = −0.14, *P*_FDR_ = 3.60 × 10^–03^; MD: 0.13, 1.50 × 10^–03^; ISOVF: 0.119, 5.0 × 10^–03^). Four traits were related to WMH, amongst which were FA (*rg* = −0.51; *P*_FDR_ = 6.60 × 10^–06^), MD (*rg* = 0.45; *P*_FDR_ = 2.30 × 10^–05^), ICVF (*rg* = −0.53; *P*_FDR_ = 0.012), and ISOVF (*rg* = 0.41; *P*_FDR_ = 1.16 × 10^–04^).

**Fig. 7:**
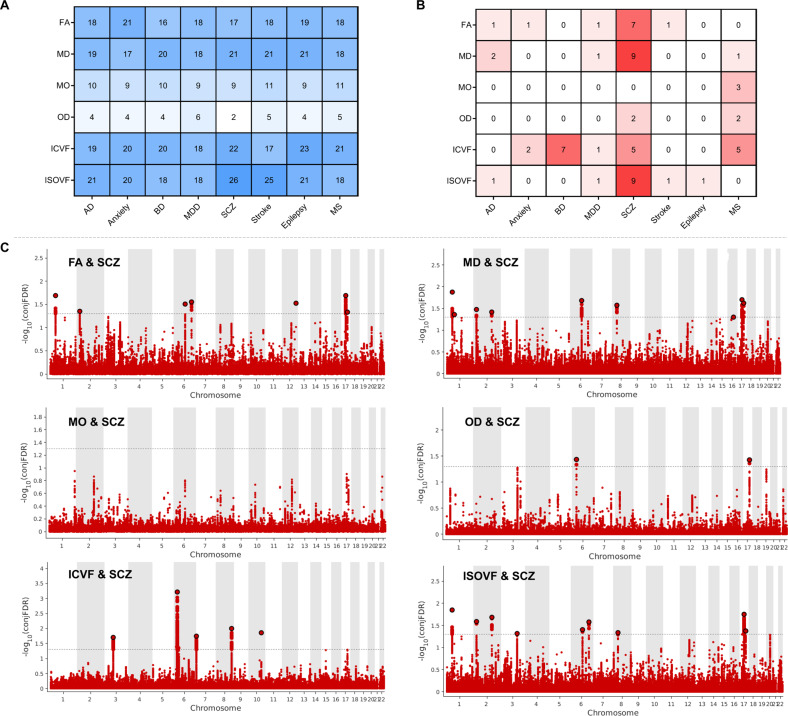
Genetic shared loci between fornix white matter microstructure and eight brain disorders. **A** Enhanced discovery of genetic loci for each of the six fornix traits when condFDR analyses were run for each of the six traits conditioned on the eight brain disorders. **B** ConjFDR analysis detected shared genetic loci across the six fornix traits and the eight clinical conditions. **C** For SCZ and the six fornix traits, conjFDR Manhattan plots are shown, illustrating the −log_10_ transformed conjFDR values for each SNP on the *y*-axis and chromosomal positions along the *x*-axis. The dotted horizontal line represents the threshold for significant shared associations (conjFDR < 0.05). Independent lead SNPs are encircled in black. FA fractional anisotropy, MD mean diffusivity, MO diffusion tensor mode, OD orientation dispersion index, ICVF intra-cellular volume fraction, ISOVF isotropic or free water volume fraction, AD Alzheimer’s disease, BD bipolar disorders, MDD major depression disorders, SCZ schizophrenia, MS multiple sclerosis, condFDR conditional FDR, conjFDR conjunctional FDR.

We leveraged the genetic overlap to discover more genetic underpinnings of fornix white matter microstructure by employing condFDR statistics with eight disorders: AD, anxiety disorders, BD, major depressive disorder (MDD), SCZ, stroke, epilepsy, and MS (Table S18). The conditional Q–Q plots indicated successive increments of SNP enrichment, consistent with polygenic overlap across fornix traits and SCZ. We discovered a total of 21 genetic loci for FA, 21, 11, 6, 23, and 26 loci for MD, MO, OD, ICVF, and ISOVF, separately (Fig. 7A). We further performed conjFDR analysis, which enables the detection of genetic loci shared between traits (Table S19). ConjFDR analysis revealed several shared loci between the six fornix traits with the eight disorders (Fig. 7B). Strikingly, we also identified 7 loci significantly overlapping between SCZ with FA, 9 loci with MD, 2 with OD, 5 with ICVF, and 9 loci significantly overlapping with ISOVF (Fig. 7C).

## Discussion

Using brain dMRI data from 30,832 UKB white British participants, this is the first large-scale GWA analysis of fornix white matter microstructure, identifying 20 independent genetic loci across 14 chromosomes. *GMNC* and *NUAK1* were found to be closely related to the fornix. Bioinformatics analyses revealed the enrichment of biological pathways of cell development and differentiation, as well as astrocyte-specific functional enrichments. Findings also highlighted the importance of fornix in brain disorders, especially in SCZ. Together, these results shed light on the genetic architecture of fornix, its biological functions, and the possibly important roles in common brain disorders.

The fornix is the predominant bundle of efferent fibers connecting the hippocampus to other brain structures [2, 48] and one of the key regions controlling memory and executive functions [49]. This is the first large-scale GWAS of fornix white matter microstructure by revealing 20 unique genomic loci associated with the 6 traits. Five of the 20 genetic risk loci were associated with only one trait (4 of them were associated with ICVF, and one with FA), suggesting that these six different indicators might have different genetic basis. DTI is the most commonly used model to measure water diffusion in the fornix tissue, providing an opportunity to detect subtle abnormalities in white matter [50]. Generally, the reduced white matter FA value of a neural structure represents a decrease in the neural structure’s microstructural integrity [51], whereas increased MD may indicate immaturity or degeneration of this region. However, the conventional DTI model does not take into account restricted and hindered diffusion [13]. The NODDI model makes up for that by determining changes in the histological compartments. NODDI provides OD, ICVF, and ISOVF maps that reflect the morphology of axons and dendrites and their branching complexity [52]. OD could detect water diffusivity [13]. The higher ISOVF indicated increased extracellular water volume, expected in neuroinflammatory states [14]. The differences in GWAS discovered loci among different metrics suggest that future research should not only focus on traditional DTI indicators but also pay more attention to other more complex measures of white matter microstructure, which may provide more detailed information to researchers and clinicians. We highlighted the *GMNC* and *NUAK1* gene, which were found in UKB and replicated in ABCD. *GMNC* has been identified as an essential regulator of axon guidance and ephrin signaling and is also involved in neuronal plasticity and regulation of gene expression [53, 54]. Recently, *GMNC* was further identified as loci for cerebrospinal fluid (CSF) phosphorylated Tau levels [55–58], and was also associated with lateral ventricular volume [55]. *NUAK1*, one of the AMP-activated protein kinase (AMPK)-related kinases [59], was reported to play an important role in regulating tau levels, indicating that *NUAK1* to be a novel therapeutic entry point for tauopathies [60].

We employed many strategies to annotate SNPs to genes, and then genes to functions. As is known to all, genetic variants associated with common diseases are usually located in noncoding parts of the human genome [61]. Positional mapping showed that most of these independent significant SNPs were significantly enriched for noncoding regions, and to be more specific, within regulatory regions. Therefore, describing the complete functional noncoding elements, as well as exploring their biological roles, is of crucial importance. Three SNPs had CADD scores > 12.37, indicating themselves as pathogenic. For example, the nearest gene of rs140589730 is *LPAR1*, which was reported to participate in regulating cell proliferation, migration, survival, and apoptosis, and could cause neurodevelopmental disorders and neuropsychiatric diseases [62]. A list of 11 genes was supported by all four mapping methods. *GNA12* was reported to play a key role in the genetic architecture underlying normal gray matter density variation in frontal and parietal regions [45] and was also a risk gene for SCZ [46]. *TIE1* could control angiopoietin function in vascular remodeling and inflammation [63]. Overexpression of circ*STAG1* could notably attenuate astrocyte dysfunction and depressive-like behaviors in mice models [64]. The functions of other genes have been reported mostly in cancer, but rarely in brain disorders. M*LLT10* (also known as *AF10*) [65] and *SKIDA1* [66] are commonly observed in acute leukemias and are indicative of a poor prognosis. Upregulation of the long noncoding RNA *CASC10* could promote cisplatin resistance in high-grade serous ovarian cancer [67]. The oncogenic role of *CDC20* in a variety of human malignancies was reported [68]. Whether these genes are involved in the development of brain disorders, and by what pathways and mechanisms, are still not clear, and hopefully future research can fill in the gap.

Using the LDSC regression method, we revealed significant genetic correlations between fornix and distinct cortical measures (the area of paracentral and cuneus, the thickness of superior temporal and parahippocampal), and subcortical measures (thalamus and pallidum). These above-mentioned brain regions are significantly associated with cognitive and memory functions. The fornix and parahippocampal-cingulum are two prominent limbic white matter tracts that connect the medial temporal lobe structures to other memory-related brain structures [69]. Jang et al.’s study showed that the posterior body of the fornix has widespread connectivity to cortical and subcortical regions, such as the pre- and post-central gyri [70]. We also identified significant correlations with several DTI phenotypes, e.g., the body of the corpus callosum, genu of the corpus callosum, anterior corona radiata, and cingulum. The strong genetic basis and inner link across brain structures were revealed. The sets of identified genes showed the highest expression in astrocytes, the second common type of neuroglia cells in the fornix, followed behind oligodendrocytes [71]. The primary function of these neuroglia cells is to form myelin, maintain homeostasis, and provide support and protection for neurons amongst others [72]. Astrocyte dysfunction has proven to be a common crossroads in neurodegenerative disorders, such as AD [73], and psychiatric diseases, such as SCZ [74]. Subsequent studies with neuroimaging data across the life span are needed to validate these findings and determine whether the genetic patterns in the fornix region differ across the life cycle.

Significant genetic correlations between fornix white matter with reaction time (an assessment scale of cognitive ability) were revealed. DTI studies suggest that forniceal measures correlate with episodic memory during brain development and aging [75]. A relationship between worse performance in a test of verbal memory or recall and reduced FA in the crus of the left fornix was identified [76]. Changes in white matter parameters have been observed in many DTI studies of AD, for example, decreased FA in the right fornix [77]. Researchers have proposed a theoretical rationale—activation of the fornix with electrical stimulation—as a therapeutic target for memory modulation [78]. Prospective randomized and double-blinded human trials are ongoing to evaluate the true potential of deep brain stimulation (DBS) to rescue memory deficits [3]. Shared genetic loci between fornix white matter microstructure and SCZ were further identified by conjFDR analysis. SCZ is thought to be a neurodevelopmental disorder. White et al. reported a study that showed a reduction in fornix volume in adolescents with SCZ [79]. Verbal and spatial memory is known to be impaired in patients with SCZ. One previous study revealed a significant association between reduced FA in the fornix and visual/spatial memory impairments in patients with SCZ [80]. DTI tractography studies revealed abnormalities in WM integrity in several structures, e.g. the fornix [81]. Given that the clinical manifestations of SCZ are quite diverse, coupled with the numerous brain regions shown to be involved (i.e., prefrontal cortex, thalamus, and anterior cingulate), it has been challenging to identify the primary causes of this disorder and the mechanisms by which the fornix are involved [82]. Moreover, lesions in the fornix are also involved in the development of MS. MS patients were reported to have reduced FA in the fornices in comparison with that in healthy controls [76, 83].

DWI has some known limitations. First, it neither can reveal the direction of information flow nor can distinguish between the different fibers that constitute a pathway (e.g., excitatory vs. inhibitory) [84]. Second, much of the fornix is located in the third ventricle, inferior to the corpus callosum, and, as such, is completely surrounded by CSF. This location makes the fornix particularly difficult to image due to ever-present susceptibility artifacts [85]. Therefore, the genetic loci found for the fornix may not be specific to this site. Third, the intrinsic morphological properties of the fornix bundle determined the inconsistencies of the imaging results: (1) it is a highly curved white matter structure, making it difficult to apply tractography algorithms that rely on angular thresholds; (2) diverging fiber populations in the medial region of the fornix bundle known as the hippocampal commissure may complicate estimation of directional diffusion [86]. Last, the NODDI model itself has recently come into question as it relies on single diffusion encoding instead of spherical tensor encoding which would better quantify microscopic anisotropy.

In summary, the current study provides new insights into the genetic architecture of fornix white matter by identifying the significant genetic loci through GWAS, the functional annotation for biological processes, analyzing genetic overlap with other traits, and showing evidence for involvement in common brain disorders. Taken together, these results advanced our understanding of the genetic architecture of the fornix and shed light on further research into the neurobiological basis of its anatomy and associations with brain disorders.

## Supplementary information

Supplemenatry Figure

Supplementary Table

## Data Availability

Our GWAS summary statistics for the fornix microstructure can be accessed via a request for the corresponding author. The individual-level imaging and genetic data used in the present study are available through UKB (https://www.ukbiobank.ac.uk/) and ABCD (https://abcdstudy.org/).

## Code availability

This study used openly available software and codes, specifically PLINK (https://www.cog-genomics.org/plink/), Michigan Imputation Server (https://imputationserver.sph.umich.edu/), FUMA (https://fuma.ctglab.n/l), MAGMA (https://ctg.cncr.nl/software/magma/, also implemented in FUMA), g:Profiler (https://biit.cs.ut.ee/gprofiler/gost/), LDSC (https://github.com/bulik/ldsc/), and condFDR/conjFDR (https://github.com/precimed/pleiofdr/).
