# Supplementary material for: The genetic architecture of fornix white matter microstructure and their involvement in neuropsychiatric disorders
Source: Transl Psychiatry. 2023 May 26;13:180. doi: 10.1038/s41398-023-02475-6 (PMC10220072; doi:10.1038/s41398-023-02475-6)
Supplement: Supplementary file 1 — Supplemenatry Figure [file 41398_2023_2475_MOESM1_ESM.docx]

**Figure S1 The results of univariate GWAS**


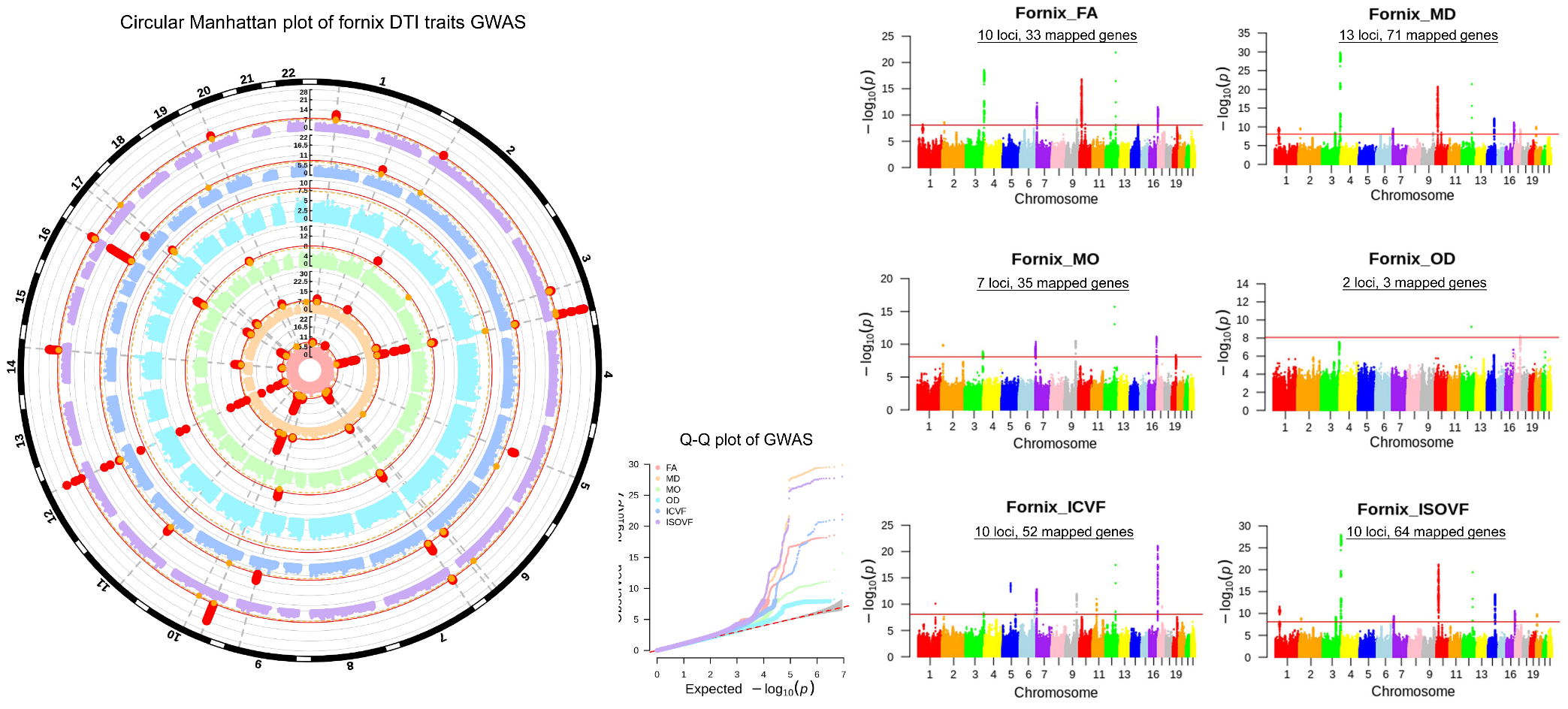


The left panel was the circular Manhattan plot of GWAS for the six fornix traits. From the periphery to the center, the plot indicates the GWAS of FA, MD, MO, OD, ICVF, and ISOVF, respectively. The orange dashed lines represent the genomic significant threshold *P* < 5 × 10^-8^, whereas the solid red lines reflect the threshold *P* < 8.3 × 10^-9^ after correction for six fornix phenotypes.

The middle panel was the Q-Q plots for six fornix white matter microstructure.

The right panel was the horizontal Manhattan plots. The red dashed lines indicate the whole-genome significance threshold of 5.0×10^-8^. At Bonferroni correction (*P* < 8.3×10^-9^) significance level, we identify 10, 13, 7, 2, 10, and 10 loci associated with FA, MD, MO, OD, ICVF, and ISOVF.

Abbreviations: GWAS, genome-wide association study; FA, fractional anisotropy; MD, mean diffusivity; MO, diffusion tensor mode; OD, orientation dispersion index; ICVF, intra-cellular volume fraction; ISOVF, isotropic or free water volume fraction.
